# A comparative analysis of outcomes of root canal therapy for pediatric medicaid beneficiaries from New York State

**DOI:** 10.3389/froh.2022.1031443

**Published:** 2022-11-19

**Authors:** Lorel E. Burns, Nihan Gencerliler, Kelly Terlizzi, Yinxiang Wu, Claudia Solis-Roman, Heather T. Gold

**Affiliations:** ^1^ Department of Endodontics, New York University College of Dentistry, New York, NY, United States; ^2^ Health Evaluation and Analytics Lab, New York University, New York, NY, United States; ^3^ Department of Population Health, New York University Langone Health, New York, NY, United States

**Keywords:** dental public health, endodontics, health services research, outcomes research, adolescent, child, population characteristics

## Abstract

**Objectives:**

This study investigated differences in the provision of root canal therapy and outcomes in a publicly insured cohort of children and adolescents.

**Methods:**

New York State Medicaid administrative claims from 2006 to 2018 were analyzed. Enrollees aged 6–18 were included in the study if they had initial non-surgical root canal therapy (NSRCT), in the permanent dentition, that allowed for at least 1 year of post-treatment follow-up. Descriptive analyses, multivariable logistic regression, and multivariable Cox proportional hazard models were used to examine the association between demographic variables (gender, age, race/ethnicity, and area-based factors) and dental treatment provision and outcomes.

**Results:**

Male gender was associated with having more than one initial NSRCT (adjusted odds ratio (aOR) = 1.06; 95% confidence interval (CI) = 1.02–1.10), as was rurality (aOR = 1.15; 95% CI = 1.06–1.24). Black/African American (AA) and Hispanic children were less likely than non-Hispanic white children to have multiple NSRCTs (aOR = 0.88; 95% CI = 0.83–0.93 and aOR = 0.78; 95% CI = 0.74–0.83). Being older or female conferred a lower hazard of an untoward event (aHR = 0.93; 95% CI = 0.92–0.94 and aHR = 0.86; 95% CI = 0.81–0.91). Compared to non-Hispanic white children, Hispanic and Black/AA children had a higher risk of untoward event (aHR = 1.31; 95% CI = 1.21–1.41 and aHR = 1.55; 95% CI = 1.43–1.67) while children of Asian descent had a lower incidence after initial NSRCT (aHR = 0.79; 95% CI = 0.71–0.88).

**Conclusion:**

Race/ethnicity was the strongest demographic predictor of provision of initial non-surgical root canal therapy, subsequent placement of a permanent restoration and the occurrence of an untoward event after NSRCT in this cohort.

## Introduction

In the United States, Medicaid is the primary source of dental insurance for children from families with low-incomes, providing dental coverage for more than 37 million children (1, 2). Medicaid provides children with dental services through the Early and Periodic Screening, Diagnostic, and Treatment Program that requires states to cover all medically necessary dental services (3). In New York State, over 1,137,566 (46.2%) children enrolled in Medicaid for at least 90 continuous days received dental services in the year 2019 (2).

Endodontic treatment, including root canal therapy, is one category of dental services covered by the Medicaid children's dental benefit. Root canal therapy may be required when a tooth's dental pulp tissue has become irreversibly inflamed, most often the result of untreated dental decay but potentially a result of dental trauma (4). Dental caries is the most common chronic disease of childhood (5). Inflammation of the pulpal tissues, resulting from dental caries, can cause intense pain that disrupts the basic activities of daily life including eating, sleeping, and participating in work or school (6, 7). Failure to stop the inflammatory process in the root canal system or significant dental trauma will lead, eventually, to pulpal necrosis and resultant infection of the root canal system (8). Neglecting treatment of the necrotic pulp through root canal therapy or tooth extraction can, in extreme cases, lead to hospitalization or death of the patient (8, 9).

There are relatively few studies of the provision of common dental procedures in pediatric populations by payer type, and they are largely focused on preventive dental procedures (10–16). A few such studies reported on differences in the average number of therapeutic dental procedures, including endodontic procedures received by children in Wisconsin (11–13), finding: (1) amongst children enrolled in a private dental insurance plan, children residing in rural and dental health provider shortage areas (DHPSAs) received higher numbers of endodontic procedures than those residing in urban and non-DHPSA areas (11, 13), and African American and Hispanic children received significantly more endodontic procedures than white children (12); (2) Child Medicaid beneficiaries received endodontic procedures at almost twice the rate of children enrolled in a private dental insurance plan and provision of endodontic procedures did not differ by DHPSA (13).

This study examines differences in treatment patterns and outcomes for one particular type of endodontic procedure, initial non-surgical root canal therapy (NSRCT), performed on permanent teeth, in children 6–18 years old enrolled in the New York State Medicaid program. We investigated differences in treatment patterns and outcomes by gender, age, race/ethnicity, poverty level, rurality, and dental health professional shortage areas (DHPSAs).

## Materials and methods

This study was a retrospective analysis of electronic insurance claims records and the enrollment database of the New York State Medicaid program. The database was comprised of 1,741,573 children and adolescents aged 6–18 who had a minimum of 1-year enrollment in the New York State Medicaid program with patient encounters that occurred between January 1, 2006, and December 31, 2018. The distribution of these individuals by race/ethnicity was as follows: non-Hispanic white (590,816; 34%); Hispanic (468,614, 27%); Black or African American (458,235, 26%); Asian (197,214; 11%); “other”/unknown (26,694; 2%). This study was approved by New York University School of Medicine's Institutional Review Board (i19-01436), expedited Category 5.

Medicaid beneficiaries were included in the study cohort if they had an initial NSRCT in the permanent dentition during their period of enrollment that allowed for at least 1 year of post-treatment follow-up. The claims dataset included patient identification number, patient age, gender, race/ethnicity, ZIP code of residence, date of treatment, procedure code for the treatment provided, tooth number treated, and date of patient disenrollment in Medicaid.

Codes on Dental Procedures (CDTs) defined by the American Dental Association and tooth number were used to identify the endodontic treatment procedure, initial non-surgical root canal therapy (NSRCT), for analysis (D3310, D3320, D3330). Further, CDTs and tooth number were used identify the placement of a permanent restoration (D2000–D2999) and/or the incidence of an untoward event after initial NSRCT. The occurrence of untoward events indicated the failure of the initial NSRCT procedure and were defined as nonsurgical re-treatment (D3346, D3347, D3348); surgical retreatment/apicoectomy (D3410, D3421, D3425); or extraction (D7140, D7210).

Census data were merged by ZIP code area to identify areas of “high poverty” and rural status, using the 2008–2012 American Community Survey of the 2012 US Census (17). Enrollees who resided in a ZIP code where more than 20% of the population lived below the federal poverty level (FPL) were classified as living in a “high” poverty area (17). Rural areas were defined according to the Federal Office of Rural Health Policy (18). For the purposes of this study, patients were classified as living in a DHPSA if their Census tract, county, or county subdivision was deemed a geographic DHPSA at any point within our study period (19). In cases where an enrollee’s ZIP code changed during the enrollment period, the ZIP code of the residence at time of initial NSRCT was used.

Data analysis was completed using SAS 9.4 (SAS Institute Inc, Cary, NC) software and R 4.0 (R Core Team, Vienna, Austria). Individuals with missing data for demographic covariates and those categorized as “other” for race/ethnicity were excluded from the analysis. Descriptive analyses were performed to examine trends in the provision of endodontic procedures. In the evaluation of treatment outcomes, initial NSRCTs were considered to be successful until the occurrence of an untoward event or censored at an identified lapse in the patient's enrollment in Medicaid. Multivariate logistic regression was used to model the association between placement of permanent restoration (yes/no) and provision of the NSRCT procedure, operationalized as a binary outcome (0 = one NSRCT; 1 = more than one NSRCT), and the following covariates: age, gender, race/ethnicity, poverty level, rurality, and DHPSA. To maintain a person-level analysis, only the first (earliest date) initial NSRCT was included in the evaluations of placement of a permanent restoration and treatment outcome. If an individual received more than one initial NSRCT on their earliest date of treatment it could not be determined which NSRCT was their “first”. Thus, these individuals were excluded. Sensitivity analyses were performed to evaluate if the exclusion of these individuals resulted in selection bias. One-way analysis of variance (ANOVA) was used to measure the association between mean time to restoration and demographic covariates for individuals with placement of a permanent restoration after initial NSRCT. Multivariate Cox proportional hazards regression was used to model the association between the aforementioned covariates and initial NSRCT survival (time to first untoward event). An additional Cox model controlled for placement of permanent restoration and tooth type. The association between type of untoward event, dichotomized to retreatment (non-surgical or apicoectomy) or extraction, and covariates was evaluated by fitting a multivariate logistic regression model. Backward variable selection was completed for both the logistic regressions and Cox proportional hazard models, based on Akaike's information criterion (AIC). A statistical significance level (alpha) of 0.05 was used throughout the analyses.

## Results

A total of 43,049 children who had NSRCTs between 2006 and 2017 were included in our sample, with a total of 65,218 NSRCTs occurring during the study period. Molars were the most commonly treated tooth type in our sample (71.4% of the procedures) and this trend persisted following stratification by demographic covariates. Characteristics of the study population are displayed in Table 1. Median patient age was 15 years (IQR: 13–16).

**Table 1:** Characteristics of study population stratified by initial root canal therapy (NSRCT) provision.

		Overall (*N* = 43,049)	One NSRCT (*N* = 29,036)	More than one NSRCT (*N* = 14,013)	*p*-value
Age category (%)	6–9	1,763 (4.1)	1,222 (4.2)	541 (3.9)	<0.001
	10–12	8,562 (19.9)	5,766 (19.9)	2,796 (20.0)	
	13–15	16,768 (39.0)	10,968 (37.8)	5,800 (41.4)	
	16–18	15,956 (37.1)	11,080 (38.2)	4,876 (34.8)	
Gender (%)	Female	23,932 (55.6)	16,289 (56.1)	7,643 (54.5)	0.002
	Male	19,117 (44.4)	12,747 (43.9)	6,370 (45.5)	
Race/Ethnicity (%)	White	16,169 (37.6)	10,464 (36.0)	5,705 (40.7)	<0.001
	Hispanic	11,953 (27.8)	8,443 (29.1)	3,510 (25.0)	
	Black	9,154 (21.3)	6,261 (21.6)	2,893 (20.6)	
	Asian	5,773 (13.4)	3,868 (13.3)	1,905 (13.6)	
High poverty (%)	No	20,487 (47.6)	13,659 (47.0)	6,828 (48.7)	0.001
	Yes	22,562 (52.4)	15,377 (53.0)	7,185 (51.3)	
Rurality (%)	Nonrural	39,731 (92.3)	26,950 (92.8)	12,781 (91.2)	<0.001
	Rural	3,318 (7.7)	2,086 (7.2)	1,232 (8.8)	
DHPSA status (%)	Non-DHPSA	38,862 (90.3)	26,111 (89.9)	12,751 (91.0)	<0.001
	DHSPA	4,187 (9.7)	2,925 (10.1)	1,262 (9.0)	

White, non-hispanic white; Black, Black or African American; DHPSA, dental health professional shortage area.

### Provision of root canal therapy

The mean number of NSRCTs per patient in this population was 1.51 with a standard deviation of 1.02 (median: 1; IQR: 1–2; range: 1–18). Table 2 reports adjusted odds ratio (aOR) estimates for the most parsimonious model of NSRCT provision, all demographic covariates analyzed had statistically significant associations with this outcome. Controlling for the other covariates, male gender was associated with having more than one NSRCT [aOR = 1.06; 95% confidence interval (CI) = 1.02–1.10], as was rurality [aOR = 1.15; 95% confidence interval (CI) = 1.06–1.24]. Black or African American and Hispanic children and adolescents were less likely than non-Hispanic white children and adolescents to have more than one NSRCT (aOR = 0.88; 95% CI = 0.83–0.93 and aOR = 0.78; 95% CI = 0.74–0.83, respectively). Children and adolescents of Asian ancestry were less likely to have had more than one NSRCT when compared to non-Hispanic white children and adolescents (aOR =  0.93; 95% CI = 0.87–0.99).

**Table 2:** Adjusted odds ratio estimates for the effect of demographic covariates on provision of more than one NSRCT from multivariate logistic regression analysis.

	Adjusted OR (95% CI)	*p*-value
Age (years)	0.98 (0.97–0.99)	<0.001
Gender (ref: female)		
Male	1.06 (1.02–1.10)	0.005
Race/Ethnicity (ref: white)		
Hispanic	0.78 (0.74–0.83)	<0.001
Black or African American	0.88 (0.83–0.93)	<0.001
Asian	0.93 (0.87–0.99)	0.028
Rural (ref: no)		
Yes	1.15 (1.06–1.24)	0.001
DHPSA (ref: no)		
Yes	0.92 (0.86–0.98)	0.016

Covariate “high poverty” excluded from model after backward variable selection.

### Placement of permanent restoration after root canal therapy

After excluding individuals that received more than one initial NSRCT at their first treatment visit, a subsample of 39,993 initial NSRCTs was available for the analysis of placement of a permanent restoration after the completion of root canal therapy. Permanent restorations were placed on 79% of teeth after initial NSRCT, with placement varying by race/ethnicity is as follows: 85% Asian; 84% non-Hispanic white; 78% Hispanic; and 71% Black/African American. In this study, all demographic covariates analyzed were significantly associated with the placement of a permanent restoration. The aOR estimates from the final, selected multivariable logistic regression model for whether or not a permanent restoration was placed after initial NSRCT is presented in Table 3. Children and adolescents of Asian ancestry were more likely to have a permanent restoration placed after initial NSRCT (aOR = 1.10; 95% CI = 1.00–1.20) compared to non-Hispanic, whites. Black or African American and Hispanic children and adolescents were less likely than non-Hispanic white children and adolescents to have a permanent restoration placed after endodontic treatment (aOR = 0.47; 95% CI = 0.44–0.50 and aOR = 0.67; 95% CI = 0.63–0.72, respectively). Those living in high poverty and rural areas were more likely to have placement of permanent restorations (aOR = 1.09; 95% CI = 1.04–1.15 and aOR = 1.13; 95% CI = 1.01–1.25, respectively). However, beneficiaries living in DHPSAs were less likely to have a permanent restoration placed after initial NSRCT (aOR = 0.88; 95% CI = 0.81–0.96). The sensitivity analysis performed to evaluate the potential of selection bias resulting from the exclusion of the aforementioned individuals from the placement of permanent restoration analysis did not result in a magnitude of change that was either clinically or statistically significant to the reported findings.

**Table 3:** Adjusted odds ratio estimates for the effect of demographic covariates on placement of permanent restoration after initial non-surgical root canal therapy (NSRCT) from multivariate logistic regression analysis.

	Adjusted OR (95% CI)	*p*-value
Gender (ref: female)		
Male	0.90 (0.85–0.94)	<0.001
Race/Ethnicity (ref: white)		
Hispanic	0.67 (0.63–0.72)	<0.001
Black or African American	0.47 (0.44–0.50)	<0.001
Asian	1.10 (1.00–1.20)	0.039
Rurality (ref: no)		
Yes	1.13 (1.01–1.25)	0.031
DHPSA (ref: no)		
Yes	0.88 (0.81–0.96)	0.003
Poverty level (ref: no)		
Yes	1.09 (1.04–1.15)	0.001

Covariate “age” excluded from model after backward variable selection.

For individuals who had a permanent restoration placed, the mean time from completion of the initial NSRCT to placement of a permanent restoration was 89 days, with a median of 21 days (range: 0–3,969 days). Individuals residing in rural areas and those of older age experienced shorter time to restoration (*p* < 0.001). There was no statistically significant difference for time to restoration by gender, race/ethnicity, residence in a high poverty area, or residence in a DHPSA.

### Treatment outcomes

After excluding individuals that received more than one initial NSRCT at their first treatment visit, a subsample of 39,993 initial NSRCTs was available for analysis of treatment outcomes. The median follow-up period following NSRCT was 46 months (IQR: 27–81). Time-to-event findings are reported at the patient-level for the first (one) initial root canal therapy completed (Table 4). Adjusted hazard ratios (aHR) are reported from the final fitted models, which excluded the DHPSA variable. Among children and adolescents undergoing initial NSRCT being older, or female conferred a lower hazard of an untoward event (aHR = 0.93; 95% CI = 0.92–0.94 and aHR = 0.86; 95% CI = 0.81–0.91, respectively). Compared to non-Hispanic white beneficiaries, Hispanic and Black or African American children had a statistically significant higher risk of untoward event (aHR = 1.31; 95% CI = 1.21–1.41 and aHR = 1.55; 95% CI = 1.43–1.67, respectively), while child and adolescent beneficiaries of Asian ancestry had a statistically significant lower hazard of untoward event after initial NSRCT (aHR = 0.79; 95% CI = 0.71–0.88). The disparities in treatment outcomes diminish but persist at statistically significant levels after controlling for placement of permanent restoration and tooth type (Table 4). The sensitivity analysis performed to evaluate the potential of selection bias resulting from the exclusion of individuals with multiple NSRCTs performed on their earliest date of the procedure did not result in a magnitude of change that was either clinically or statistically significant to the reported findings on treatment outcomes.

**Table 4:** Adjusted hazard ratio (HR) estimates for the effect of demographic covariates on occurrence of untoward event from multivariate cox proportional hazard analysis (covariate “DHPSA” excluded from model after backward variable selection).

	Adjusted HR (95% CI)	*p*-value	Model controlled for placement of permanent restoration and tooth type	
			Adjusted HR (95% CI)	*p*-value
Restoration (ref: no)				
Yes			0.23 (0.22–0.24)	<0.001
Tooth type (ref: anterior)				
Premolar			1.45 (1.21, 1.74)	<0.001
Molar			2.14 (1.84, 2.49)	<0.001
Age (years)	0.93 (0.92–0.94)	<0.001	0.93 (0.92–0.94)	<0.001
Gender (ref: male)				
Female	0.86 (0.81–0.91)	<0.001	0.83 (0.78–0.88)	<0.001
Race/Ethnicity (ref: white)				
Hispanic	1.31 (1.21–1.41)	<0.001	1.14 (1.05–1.32)	<0.001
Black or African American	1.55 (1.43–1.67)	<0.001	1.22 (1.13–1.32)	<0.001
Asian	0.79 (0.71–0.88)	<0.001	0.78 (0.71–0.87)	<0.001
Rurality (ref: no)				
Yes	1.08 (0.95–1.22)	0.239	1.14 (1.01–1.29)	0.038
Poverty level (ref: no)				
Yes	0.93 (0.88–0.98)	0.013	0.95 (0.89–1.00)	0.062

**Figure 1:**
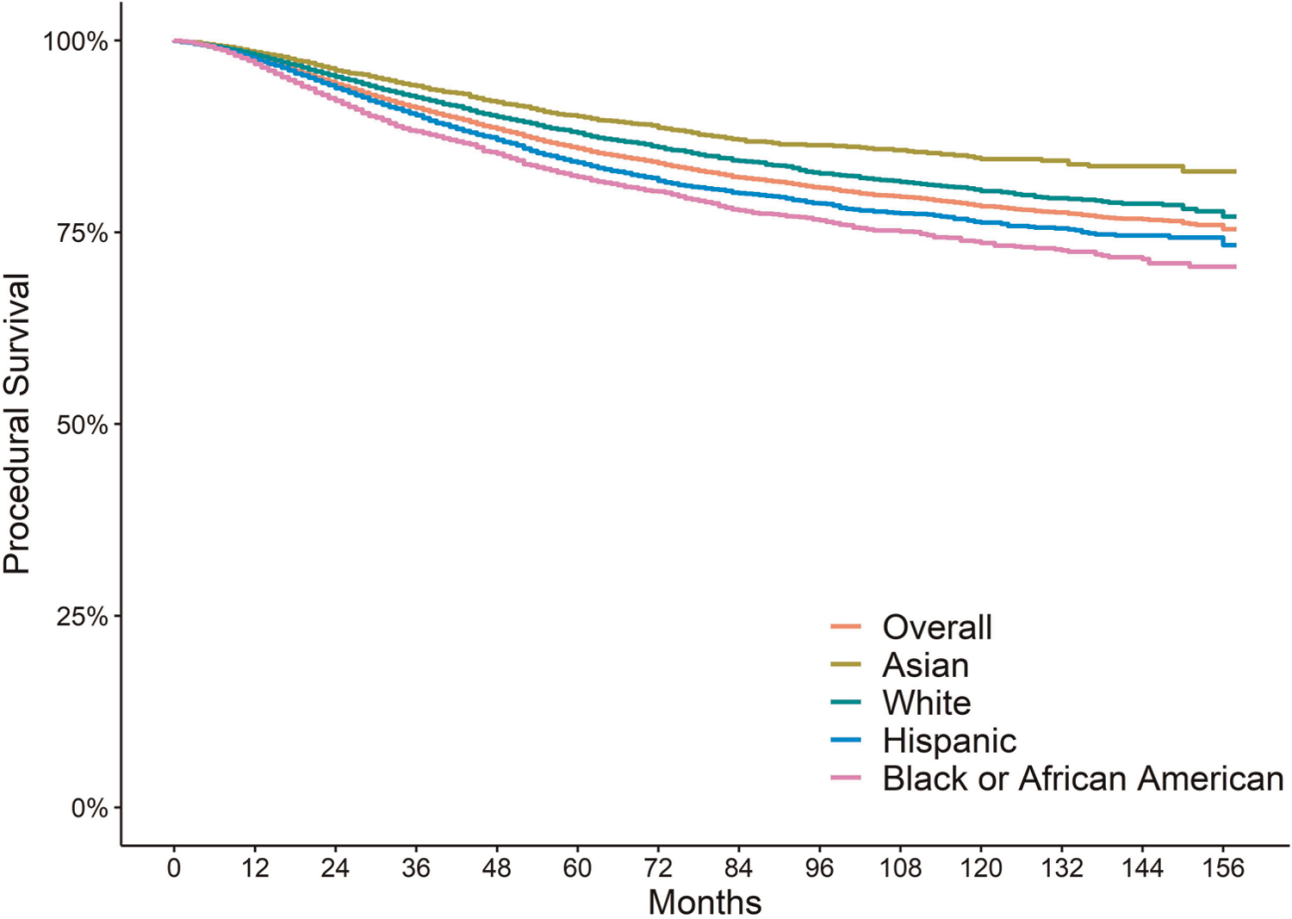
Survival of initial root canal therapy by race/ethnicity.

Out of 39,993 analyzed initial NSRCTS, 5,885 (14.71%) realized an untoward event, with a median time to untoward event of 33 months (IQR = 18–57). Overall procedural survival of initial NSRCT as well as procedural survival by race/ethnicity are depicted in Figure 1. The first untoward event after NSRCT was characterized as follows: 9.8% non-surgical endodontic retreatment; 3.0% Apicoectomy (surgical retreatment); 87% extraction. A logistic regression model fitted to the subsample of patients who realized an untoward event revealed that several covariates were significantly associated with having a tooth extraction as their first untoward event rather than endodontic retreatment (non-surgical or apicoectomy). The covariates associated, at a statistically significant level, included race/ethnicity, age, rurality, and area poverty level. Hispanic and Black or African American child and adolescent beneficiaries were significantly more likely to have a tooth extracted compared to non-Hispanic whites (aOR = 1.88; 95% CI = 1.55–2.29 and aOR = 4.12; 95% CI = 3.21–5.34, respectively). The odds of tooth extraction for children and adolescents of Asian ancestry did not differ from that of white children and adolescents (aOR = 0.99; 95% CI = 0.78–1.27). Rurality and older age were positive predictors of having a tooth extraction rather than retreatment (aOR = 2.08; 95% CI = 1.45–3.06 and aOR = 1.10; 95% CI = 1.07–1.13, respectively) while living in a “high poverty area” was negatively associated with extraction (aOR = 0.80; 95% CI = 0.68–0.95). Residence in a DHPSA and male gender were not statistically significant predictors of having a tooth extraction rather than retreatment (aOR = 1.28; 95% CI = 0.97–1.72 and aOR = 1.17; 95% CI = 1.00–1.38, respectively).

## Discussion

In this study of child and adolescent New York State Medicaid beneficiaries, the provision of initial non-surgical root canal therapy (NSRCT), the subsequent placement of a permanent restoration, and the occurrence of untoward events following initial NSRCT were associated with demographic covariates. The provision of root canal therapy was significantly associated with an individual's age, gender, race/ethnicity, residence in a rural area and/or DHPSA. Being older or female conferred a lower hazard of an untoward event after the endodontic procedure, initial NSRCT. African American or Black and Hispanic children and adolescents had a higher risk of and untoward event after initial NSRCT than that of non-Hispanic whites, while children and adolescents of Asian descent had a lower risk of an untoward event compared to non-Hispanic whites. To our knowledge, this study is the first to report racial/ethnic comparisons in the provision and outcomes of endodontic procedures for children enrolled in public payer insurance in the United States.

Overall procedural survival rates for initial NSRCT are high (20–25). Treatment outcomes for over 50,000 children enrolled in the New York State Medicaid program, inclusive of the cohort in this study, were previously analyzed at the tooth-level and the procedural survival rates were 98% at 1 year, 92% at 3 years, 88% at 5 years, and 80% at 10 years (25). The results in this manuscript are distinct from the previous publication as they extend the analysis of the New York State Medicaid cohort to evaluate the association of demographic characteristics, such as race/ethnicity, with the provision and outcomes of initial nonsurgical root canal therapy at the person-level. Studies show that the placement of a permanent restoration after the completion of initial NSRCT is associated with improved treatment outcomes (20, 25). The findings of this study suggest that permanent restoration may act as a mediator of the relationship between race/ethnicity and treatment outcomes. However, after controlling for placement of permanent restorations racial disparities reduced but persisted at statistically significant levels for the hazard of an untoward event after initial non-surgical root canal therapy. This suggests that factors beyond the placement of a permanent restoration may account for the realized disparity in endodontic treatment outcomes in this population. Beyond placement (yes/no) of a permanent restoration after endodontic treatment, time to permanent restoration influences the survival rate of initial NSRCTs (21, 25). In this study, beneficiaries residing in rural areas and those of older age experienced statistically significant shorter time to permanent restoration (*p* < 0.001). We found that children residing in rural areas were more likely to have a permanent restoration placed on the same day as the completion of the initial NSRCT procedure. This finding suggests that providers may consider travel time or distance in their decisions regarding timing for placement of permanent restorations after initial NSRCT. There were no statistically significant differences for beneficiaries residing in a DHPSA or for race/ethnicity for time to permanent restoration, suggesting that time to permanent restoration does not account for the disparities in treatment outcomes by race/ethnicity detected in this study.

When procedural failures of initial NSRCT do occur, providers and patients may consider endodontic retreatment (non-surgical retreatment or apicoectomy) or tooth extraction as subsequent treatment options. Consistent with previous studies in adult populations, extraction was the most common type of untoward event immediately following the failure of an initial NSRCT procedure in this pediatric cohort (22, 25, 26). In our analysis, we found that Hispanic and Black or African American child and adolescent beneficiaries were significantly more likely to have had a tooth extraction rather than endodontic retreatment immediately following failure of the initial NSRCT procedure, compared to non-Hispanic whites. Additionally, those living in rural areas were less likely to have a tooth-saving retreatment procedure.

A recent white paper published by the American Association of Public Health Dentistry in 2021 acknowledged racism as a dental public health crisis and noted that “it is critically important to identify actions that the dental public health community can take to reduce and eliminate racism” (27). While we do not have evidence that racism is contributing to the realized differences in the provision of endodontic treatment and outcomes by race/ethnicity in this present study, we believe that explanations for the realized differences in the receipt of endodontic treatment and treatment outcomes by racial and ethnic groups should be explored. For example, findings from a recent randomized control trial reported that dentists in Italy were more likely to recommend root canal treatment for white patients compared to Black patients (28). Additional studies in the dental literature have examined racial differences in the receipt of endodontic procedures but few have been conducted in pediatric populations (11, 29–31). These findings have implications for existing biases in endodontic treatment planning, which may influence the provision of endodontic treatment. Other explanations for the differences in the provision of endodontic treatment and procedural outcomes by race/ethnic groups in this study may include the time or frequency at which dental care is pursued. Differences in timing may influence disease progression, inclusive of tooth restorability. This may in turn impact treatment planning and treatment outcomes. Another consideration may be differences in parental attitudes or beliefs about endodontic treatment or the importance of maintaining the natural, permanent dentition. Behavioral differences and oral health literacy may influence whether endodontic treatment is pursued when presented by the dentist as a treatment option. Future studies may explore factors related to provider, patient, and caretaker perceptions of endodontic treatment or replicate this research methodology amongst pediatric public-payer beneficiaries of a different state, to see if the reported demographic associations persist in other geographic areas.

A salient strength of this study is the large size of the sample and range of years included in the analysis. This allows for a more comprehensive assessment of trends and policy implications across the study cohort. The primary limitation of this study is the non-clinical nature of the data. It is possible that some administrative claims reports are missing, or details are unaccounted for, such as diagnoses or differences in patient/provider preferences or behaviors. An additional limitation of this study is that the slight variations in the reported differences in the distribution by race/ethnicity between all enrolled children and adolescents in NY State Medicaid and those included in this study cannot be explained by the reported results. Possible reasons for these variations in distribution may include differences in the need for endodontic treatment and the level of acceptance of endodontic treatment versus tooth extraction between racial/ethnic groups.

The results of this study may be used by policy makers and clinicians seeking to increase understanding of the association between demographic factors and the provision of dental treatment and outcomes amongst pediatric Medicaid beneficiaries. Awareness and acknowledgement of these associations may lead to implementation of practices that can reduce oral health disparities.

## Data Availability

The original contributions presented in the study are included in the article/Supplementary Material, further inquiries can be directed to the corresponding author/s.
